# Supplementary material for: Long Term Outcome of Surgical Treatment of Central Introital Dyspareunia
Source: J Clin Med. 2022 Apr 7;11(8):2066. doi: 10.3390/jcm11082066 (PMC9027974; doi:10.3390/jcm11082066)
Supplement: Supplementary file 1 [file jcm-11-02066-s001.zip › jcm-1528728-supplementary.pdf]

rz tienen

English version

1. Date of birth _ ____-_____-_____

2. Length _ ____ m

3. Weight __ ___ kg

4. Do you have one of the following diseases?
Diabetes?
Heart or vascular disease
Lung disease
None of them

5. Do you smoke?
No.
Yes, _____ number of cigarettes per day

6. Do you have smoked in the past?
No.
Yes, from _____ (age) to _____ (age)

7. Gravidity, have you been pregnant and given birth naturally before the procedure? Yes/ No
If yes, number of natural births (caesarean sections not included)?
……

8. Gravidity, have you been pregnant and gave birth after the procedure? Yes/ No
If yes, number of natural (vaginal) births? ……………………………
Number of caesarean sections? ………………………………………………

How long was the interval between the procedure (vulvoplasty) and the first natural (vaginal) birth (in months) (cesarean section not included):……………………………months

Euro-Qol 5D Questionnaire

For the questions below, please circle the number that suits you best.

Mobility:
1. I have no problems with mobility
2. I experience limitations for some movements
3. I am bedridden

Self-care:
1. I have no problems taking care of myself
2. I need help washing or dressing myself
3. I cannot wash or dress myself

Daily activities: (eg work, studies, household, leisure activities, etc.)
1. I have no problems exercising my daily activity
2. I need help with my daily activity
3. I am completely dependent on a second person for my daily activity

Pain / discomfort:
1. I have no pain or other discomfort
2. I have moderate pain or other discomfort
3. I suffer from extreme pain or other serious discomfort

Anxiety / Depression:
1. I am not anxious or depressed
2. I have moderate anxiety or depressive feelings
3. I feel extremely anxious or seriously depressed

Questions about the procedure and the course during and immediately after the operation

During the operation

Experience during the procedure
1.Very painful
2. Painful
3. Tolerable
4. Virtually no pain

Did you have problems with the operating wound after the surgery	Yes<br>No (you can skip the following question)
If so, what was your issue? (Multiple answers are possible)	Wound was not healed after 2 weeks<br>Infection, treated with antibiotics<br>Infection, for which hospital readmission and / or surgery was needed<br>Other, ____________________

Long-term follow-up
1. Have any treatment (s) taken place after the first intervention in …… ..? (multiple answers are correct)

1 - not painful, 2 - tolerable, 3 - painful 4 - very painful 5 - not possible (no penetration possible)

rz tienen

Beoordeling	1 (not painful)	2	3	4	5 (not possible because of the pain)
1 month after the procedure					
6 month after the procedure					
12 month after the procedure					
24 month after the procedure					
More than 2 years after the procedure					

3. Characteristics of current complaints with sexual intercourse:
O Always pain, even without coitus
O Pain when sitting, cycling
O Penetration pain
O When moving back and forth (sanding)
O During orgasm
O Abdominal pain
O Burning AFTER having sex (including peeing)

4. How long have you had (again) complaints with coitus:
O <6 months
O <1 year
O 1-3 years
O 3-5 years
O> 5 years

5. Frequency of coitus
Number of times having intercourse per week: …… per month: …… per year: …….
How often do you have to stop during intercourse because of the pain: ...% of attempts
Is your libido also affected in a negative direction: yes / no
Last time penetration:… .. weeks / months / years ago (circle)

6. Severity of the pain problem in your daily life
(not at all serious) 0 1 2 3 4 5 6 7 8 9 10 (very disabled)

7. What do you think is the cause of pain during intercourse? (Check all that apply)
O Not applicable because I am no longer in pain
O Intimacy problems or relationship problems
O No desire, problems with excitement or lubrication
O Fear of the sexual experience
O Pelvic pain (endometriosis, inflamed pelvis) disease, irritable bowel syndrome)
O Vaginismus (painful spasms of the vaginal muscles with attempted penetration)
O Vulvar pain or vulvodynia (pain due to external genitalia such as labia)
O Other (please describe): ………………………………………………………………………………… ..

8. In the last 4 weeks, how often did you experience a feeling or pain with vaginal penetration?
O I have had no coitus (no penetration)
O Almost always or always
O Usually or more than half the time
O Sometimes (1/2 of the time)
O Several times
O Almost never

9. In the last 4 weeks, what degree of discomfort or pain during vaginal penetration would you indicate?
O I have had no coitus (no penetration)
O Very high
O High
O Medium
O Low
O Very low or no discomfort

Decision
Would you recommend the procedure to a friend after a correct diagnosis / indication?
Yes/ No

If not, why?
……………………………………………………………………………………… ..
